# Mutant p53 promotes RCP-dependent chemoresistance coinciding with increased delivery of P-glycoprotein to the plasma membrane

**DOI:** 10.1038/s41419-021-03497-y

**Published:** 2021-02-24

**Authors:** Vinaya Phatak, Yannick von Grabowiecki, Justyna Janus, Leah Officer, Caron Behan, Lydia Aschauer, Lucia Pinon, Hannah Mackay, Sara Zanivan, Jim C. Norman, Michael Kelly, John Le Quesne, Patricia A. J. Muller

**Affiliations:** 1 MRC Toxicology Unit, University of Cambridge, Cambridge, UK; 2 Cancer Research UK Manchester Institute, University of Manchester, Manchester, UK; 3 Centre for Core Biotechnology Services, University of Leicester, Leicester, UK; 4 Cancer Research UK, Beatson Institute, Glasgow, UK; 5 Institute of Cancer Sciences, University of Glasgow, Glasgow, UK; 6 Leicester Cancer Research Centre, University of Leicester, Leicester, UK; 7 Present Address: Avacta Life Sciences, Cambridge, UK; 8 Present Address: Institute of Cancer and Genomic Sciences, University of Birmingham, Birmingham, UK

**Keywords:** Tumour-suppressor proteins, Preclinical research

## Abstract

TP53 is the most frequently mutated gene in cancers. Mutations lead to loss of p53 expression or expression of a mutant protein. Mutant p53 proteins commonly lose wild-type function, but can also acquire novel functions in promoting metastasis and chemoresistance. Previously, we uncovered a role for Rab-coupling protein (RCP) in mutant p53-dependent invasion. RCP promotes endosomal recycling and signalling of integrins and receptor tyrosine kinases. In a screen to identify novel RCP-interacting proteins, we discovered P-glycoprotein (P-gp). Thus, we hypothesised that mutant p53 could promote chemoresistance through RCP-dependent recycling of P-gp. The interaction between RCP and P-gp was verified endogenously and loss of RCP or mutant p53 rendered cells more sensitive to cisplatin and etoposide. In mutant p53 cells we detected an RCP-dependent delivery of P-gp to the plasma membrane upon drug treatment and decreased retention of P-gp substrates. A co-localisation of P-gp and RCP was seen in mutant p53 cells, but not in p53-null cells upon chemotherapeutic exposure. In conclusion, mutant p53 expression enhanced co-localisation of P-gp and RCP to allow for rapid delivery of P-gp to the plasma membrane and increased resistance to chemotherapeutics.

## Introduction

During the acquisition of chemoresistance, many cancer cells upregulate the expression of transporters mediating drug efflux^1,2^. Research has therefore focussed on strategies to oppose efflux transporter function^3,4^. Of these transporters, P-glycoprotein (P-gp, MDR1, gene: *ABCB1*) has received most attention, and overexpression, stabilisation as well as polymorphisms in the *ABCB1* gene are associated with chemoresistance^4–6^. P-gp is involved in efflux of a range of cellular toxins, and also chemotherapeutic drugs^7,8^.

p53 is a tumour suppressor involved in controlling the balance between cell survival and death upon stresses, including chemotherapeutics^9^. p53 is frequently mutated in a variety of cancers, leading to loss of protein expression or expression of a mutated protein. Many mutant p53 proteins acquire gain-of-function properties promoting cell proliferation, metastasis and chemoresistance^10^. In cells, mutant p53 causes resistance against chemotherapeutics and mutant p53 expression can be predictive for patient response to chemotherapy^11–18^. These gains-of-function are often the result of mutant p53 interfering with other p53 family members^10,19^. Previously, we showed that mutant p53 inhibits p63 and thereby promoting Rab-coupling protein (RCP)-dependent invasion and metastasis^20,21^. Mutant p53 promoted the interaction between RCP and integrins and facilitated EGFR signalling to ERK1/2 and Akt. In a screen to detect novel RCP-interacting proteins, we discovered P-gp. P-gp has been shown to translocate from the Golgi complex to the plasma membrane in response to chemotoxic drugs through regulation via Rab GTPases^22–25^. However, it is unknown to what extent this translocation actually plays a role in chemoresistance.

Here, we describe a novel function for RCP in promoting drug-dependent trafficking of P-gp to the plasma membrane in mutant p53-expressing cancer cells. These findings are important because RCP-regulated trafficking is now established to be a major hub in mutant p53’s pro-invasive gain-of-function. These findings therefore offer a basis for the development of RCP-inhibiting drugs that may simultaneously impair invasiveness and restore chemosensitivity.

## Results

### Loss of RCP in mutant p53-expressing cells increases sensitivity to chemotherapeutics

**Fig. 1:**
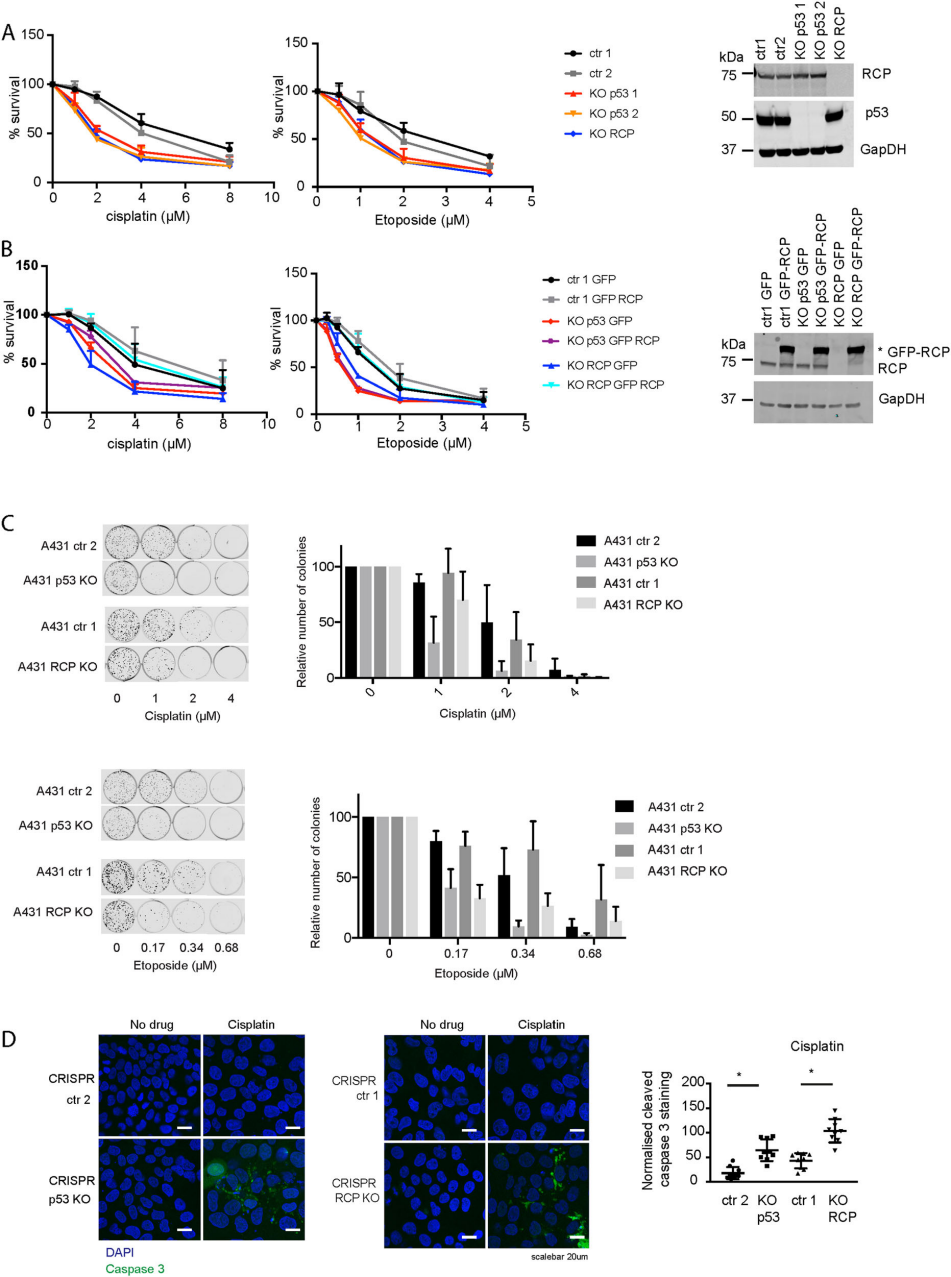
Chemoresistance in A431 cells is dependent on RCP and mutant p53 expression. **A** Resazurin survival assay of two different ctr A431 cells, two different p53 KO cells and RCP KO A431 cells in response to increasing doses of cisplatin and etoposide, measured after 96 h. Statistical differences were measured using a paired two-way ANOVA corrected for multiple testing (two-sided) and IC50 values were measured using a linear regression analysis. IC50 and *P*-values can be found in Supplemental Tables 1 and 2. Error bars indicate SD of average values of three independent experiments with three technical repeats each. Western blot panels are shown on the right incubated for RCP, p53 and GAPDH as loading control. **B** Resazurin survival assay of ctr, p53 KO cells and RCP KO A431 cells that were selected for GFP or GFP-RCP expression upon transfection. Survival was measured 72 h after increasing doses of cisplatin of etoposide, measured after 96 h. Statistical differences were measured using a paired two-way ANOVA corrected for multiple testing (two-sided) and IC50 values were measured using a linear regression analysis. IC50 and *P*-values can be found in Supplemental Tables 3 and 4. Error bars indicate SD of average values of three independent experiments with three technical repeats each. GFP-RCP expression was verified using western blot (right panels) using RCP and GAPDH as loading control. **C** A431 ctr1 and ctr2, p53 KO and RCP KO cells were treated with increasing amounts of cisplatin (top) or etoposide (bottom), allowed to grow in colonies (left panel) and quantified (right panel). Statistical differences were measured using a paired two-way ANOVA corrected for multiple testing (2-sided). *P*-values can be found in Supplemental Table 5. Error bars indicate SD of average values of three independent experiments with three technical repeats each. **D** Immunofluorescence and confocal imaging of cleaved caspase 3 (CC3) in A431 ctr1 and ctr2, RCP KO and p53 KO cells upon treatment with cisplatin for 48 h (4 µM). Automated quantification of CC3 staining per DAPI measured using fluorescence microscopy is shown on the right in a box and whiskers plot, with mean values indicated. Error bars indicate SD of three independent experiments. * Indicates statistical significance tested using a *t*-test, two-sided *P* < 0.0001 for both.

Previously, we demonstrated that mutant p53 drives invasion and metastasis by regulating RCP-dependent recycling of growth factors and integrins. Another gain-of-function of mutant p53 is chemoresistance and we therefore determined if RCP promotes mutant p53-dependent chemoresistance. We used A431 mutant p53 R273H-expressing cells and generated CRISPR control (ctr, 2 different clones), p53 CRISPR knockout (p53 KO, 2 clones generated from different guide strands) and RCP CRISPR knockout cells (RCP KO). Next, we investigated their sensitivity to cisplatin and etoposide using survival assays. Loss of RCP or p53 decreased cell survival upon cisplatin and etoposide exposure (Fig. 1A, Supplemental Tables 1 and 2). Overexpression of GFP-RCP in RCP KO cells restored survival upon cisplatin and etoposide exposure, indicating a specific role for RCP in driving chemoresistance (Fig. 1B, Supplemental Tables 3 and 4). However, overexpression of GFP-RCP in p53 KO cells did not restore survival, suggesting that mutant p53 is not regulating RCP expression levels, but rather a regulatory pathway that drives RCP function.

Consistent with a decreased cell survival of p53 KO and RCP KO cells, we also noted a decreased potential to form colonies upon cisplatin or etoposide exposure (Fig. 1C). Cisplatin also caused a dose-dependent increase in cleaved caspase 3 (CC3) that was more pronounced in RCP KO and p53 KO cells (Fig. 1D and Supplemental Table 5).

Cisplatin and etoposide resistance could also be observed in the colon carcinoma cell line HCT116 that expresses mutant p53 R248W compared to HCT116 p53-null cells (Supplemental Fig. 1A and Supplemental Table 10). Cisplatin resistance was RCP-dependent as RCP siRNA reduced cell viability in response to cisplatin exposure (Supplemental Fig. 1B and Supplemental Table 10). Similarly, MDA-MB-231 cells that express a mutant p53 R280K, showed an RCP-dependent resistance to cisplatin (Supplemental Fig. 1C and Supplemental Table 10). These data suggest that mutant p53 regulates chemoresistance in an RCP-dependent manner.

**Fig. 2:**
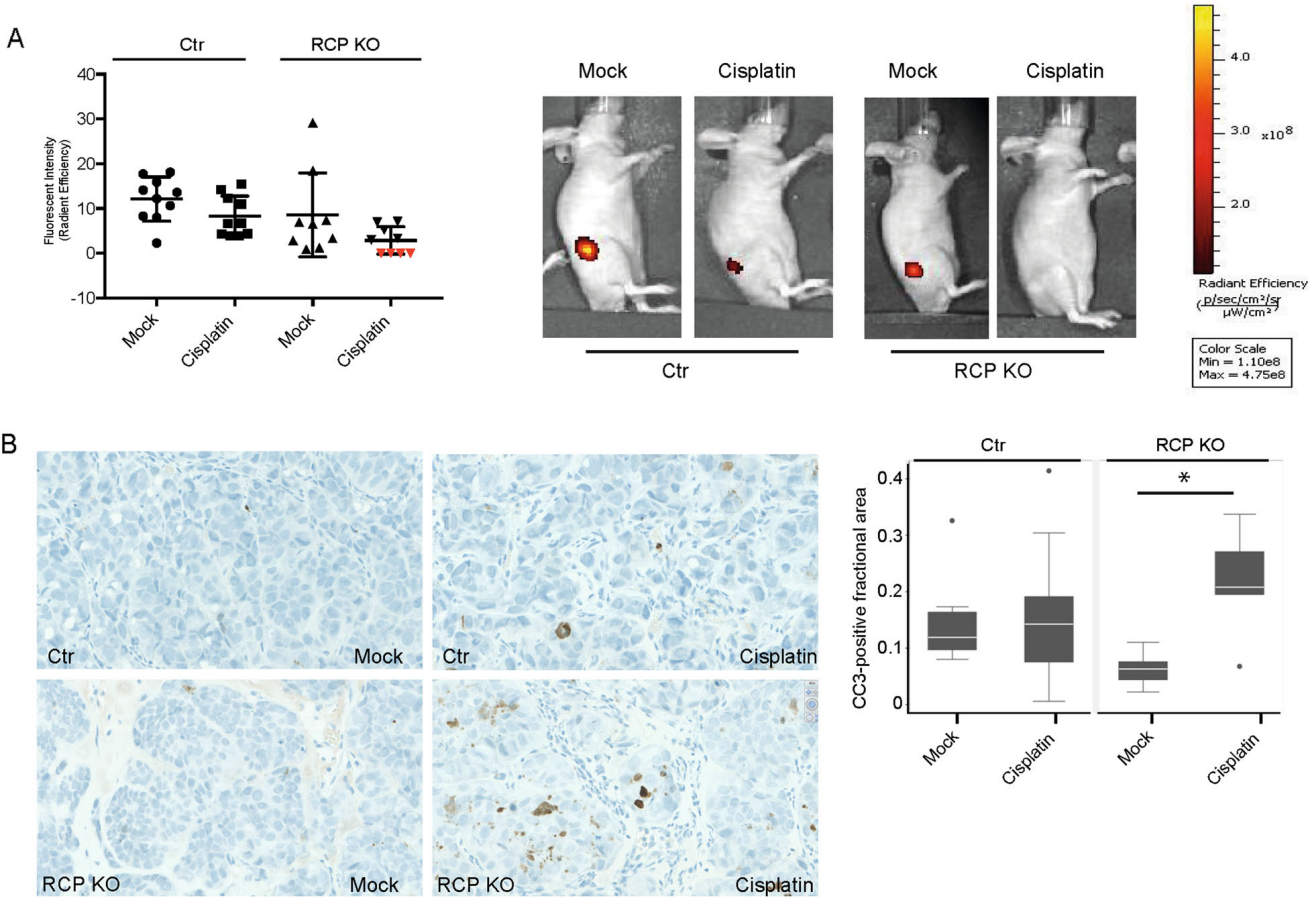
RCP KO cells are more sensitive to cisplatin in vivo. **A** mCherry-expressing A431 control and A431 RCP KO cells were injected subcutaneously and treated with cisplatin (cisplatin 5 mg/kg, intraperitoneally) or a vehicle control. The tumour size was inferred using fluorescence and quantified using the IVIS software. Error bars indicate SD. Red triangles indicate no measured signal and therefore measurements were set to 0. Representative images of iRFP signals in mice are shown in the right panels. **B** Cleaved caspase-3-positive areas were determined in each tumour and representative images are shown in the left panels, and plotted for each group in the right panel. Error bars indicate SD and * indicates a statistically significant difference (*P* = 0.01). Differences between growth rates and fractional cleaved caspase-positive areas were assessed by Mann-Whitney two-sample statistic with Stata SE 13.1. Scale: 40X.

To determine whether loss of p53 or RCP promotes cisplatin sensitivity in vivo, we generated fluorescent mCherry-expressing A431 cells and injected these subcutaneously in mice. Tumours were palpable within 2 weeks after injections. Unexpectedly, no tumours with loss of p53 expression were detected. Loss of RCP was confirmed in RCP KO tumours, which were significantly smaller than A431 control tumours (Fig. 2A and Supplemental Fig. 2A). Given that proliferation in culture was not affected in RCP KO cells and p53 KO cells (Supplemental Fig. 2B), these data could indicate that the knockout cells lack cancer stem cell-like activity as reported by others^26–28^. Similarly, we observed that A431 cells grow fewer numbers and smaller colonies in anchorage-independent assays upon loss of RCP or mutant p53 expression (Supplemental Fig. 2C and Supplemental Table 11).

Despite the initial growth differences, we next investigated whether tumours with loss of RCP were more sensitive to cisplatin treatment. All mice in the control group with (10/10) or without cisplatin (10/10) developed tumours. Mice that had received RCP knockout cells all developed tumours as well (9/9), but in this group only 5 out of 9 mice that were exposed to cisplatin developed tumours and were measurable by fluorescence (Fig. 2A and Supplemental Fig. 2D). Cisplatin treatment of RCP KO tumours resulted in the smallest tumours that were whiter in appearance when dissected (Supplemental Fig. 2D). Compared to cisplatin treated control tumours, RCP KO tumours responded about 1.5-fold more to cisplatin than control tumours, although this was not significant due to high variation (Fig. 2A). As we detected an increase in CC3 expression in RCP KO cells compared to control cells upon cisplatin treatment (Fig. 1D), we next investigated CC3 levels in the xenograft tumours. RCP KO tumours treated with cisplatin had a significantly larger number of CC3-positive cells than untreated tumours, while no difference was observed in CC3 levels in control tumours treated with or without cisplatin (Fig. 2B).

### RCP interacts with P-gp

**Fig. 3:**
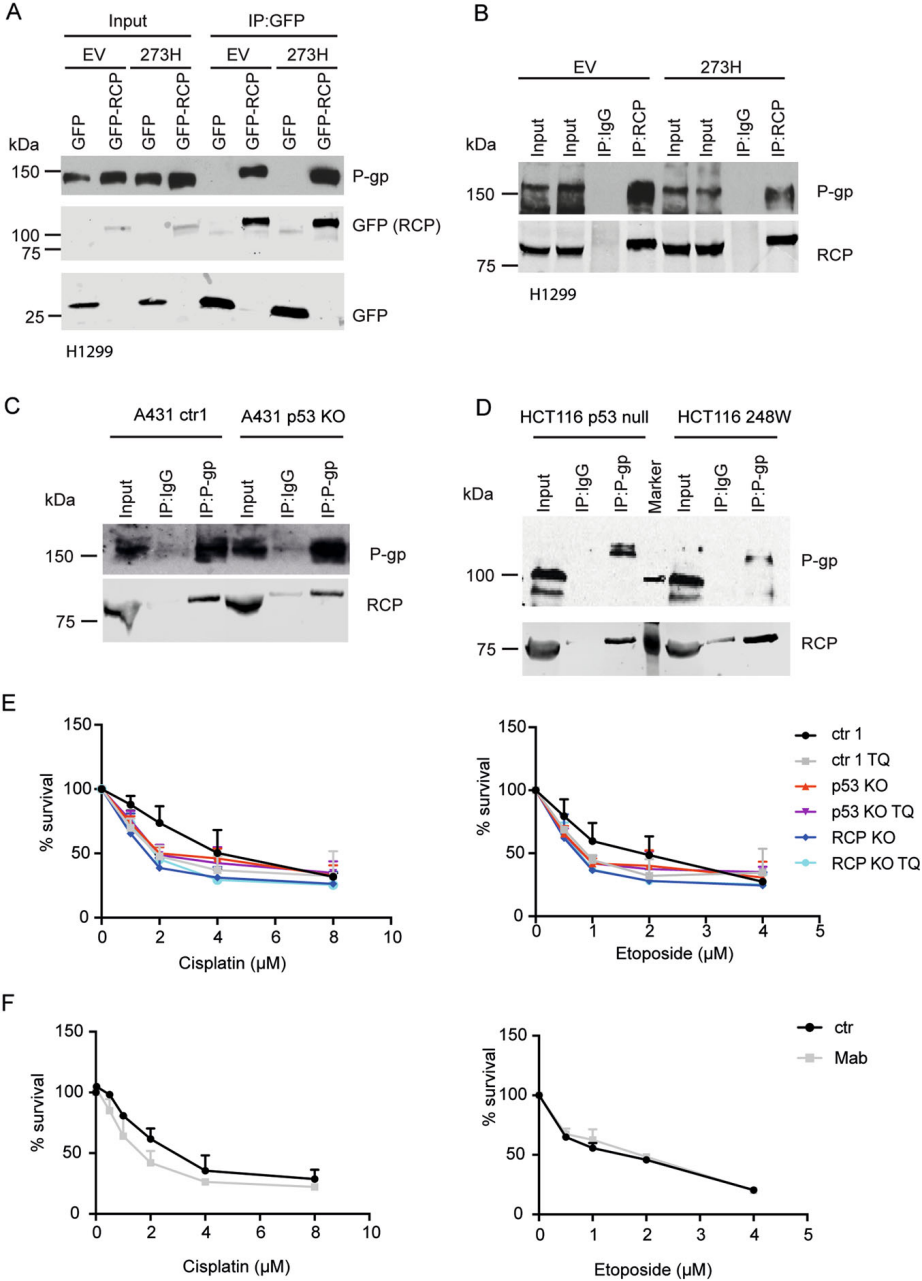
RCP interacts with P-gp. **A** H1299 cells, stably expressing mutant p53 R273H or an empty vector (EV) (PCB6) were transfected with GFP or GFP-RCP and cell lysates were immunoprecipitated with GFP. P-gp and GFP expression was analysed by western blot. **B** Cell lysates of H1299 cells stably expressing mutant p53 R273H or an EV (PCB6) were immunoprecipitated with RCP or an IgG control. P-gp and RCP expression was analysed by western blot. **C** P-gp and IgG were immunoprecipitated from A431 ctr or A431 KO cells. Endogenous P-gp and RCP protein levels were analysed by western blot. **D** P-gp and IgG were immunoprecipitated from HCT116 -/- or 248W cells. Endogenous P-gp and RCP protein levels were analysed by western blot. **E** Resazurin survival assay of ctr, p53 KO or RCP KO cells treated with increasing doses of cisplatin and etoposide in combination with 0.5 µM Tariquidar or DMSO control for 96 h. Statistical differences were measured using a paired two-way ANOVA corrected for multiple testing (two-sided) and IC50 values were measured using a linear regression analysis. IC50 and *P*-values can be found in Supplemental Tables 6 and 7. Error bars indicate SD of average values of three independent experiments. **F** Resazurin survival assay of ctr1 A431 cells treated with increasing doses of cisplatin or etoposide in the presence or absence of Mab16 (1 µg/mL) for 96 h. Statistical differences were measured using a paired two-way ANOVA (two-sided) corrected for multiple testing and IC50 values were measured using a linear regression analysis. IC50 and *P*-values can be found in Supplemental Table 8. Error bars indicate SD of average values of three independent experiments.

Previously, we performed a mass spectrometry proteomic screen to identify RCP interaction partners in H1299 cells^21^. We detected an interaction between RCP and P-gp that was more pronounced in mutant p53 R273H cells than in control empty vector cells. This interaction was validated by immunoprecipitation of GFP-RCP from GFP-RCP-transfected mutant p53 and p53-null H1299 cells (Fig. 3A), and again suggested a slightly better interaction between RCP and P-gp in mutant p53 R273H cells. The interaction was validated endogenously using an RCP pulldown (Fig. 3B), although an increased interaction in mutant p53 cells was not observed in this experiment.

An endogenous interaction between RCP and P-gp was also observed in A431 cells and in HCT116 cells (Fig. 3C, D). As endogenous P-gp is not easily detected by western blot, it is harder to make conclusions as to whether P-gp is interacting stronger with RCP in mutant p53-expressing cells than in p53-null cells. However, in A431 cells, the RCP co-immunoprecipitation with P-gp appears weaker than in p53 KO cells (Fig. 3C). In HCT116 cells, less P-gp is immunoprecipitated by P-gp antibodies in mutant p53-expressing cells, compared to p53-null cells, although it was difficult to see P-gp expression in the input. However, co-immunoprecipitation of RCP by P-gp resulted in a stronger RCP signal in mutant p53-expressing cells, compared to p53-null cells (Fig. 3D). Together, these results point to P-gp being an interaction partner of RCP. Our results might indicate that the RCP/P-gp interaction is more pronounced in mutant p53-expressing A431 and HCT116 cells than in p53-null cells. However, it has to be noted that it is difficult to analyse this interaction in detail. P-gp is a membrane protein that requires stringent lysis buffer to resolve for western blot, but such conditions are unfavourable for maintaining the interaction during immunoprecipitation.

To prove that P-gp is important in mutant p53 and RCP-mediated chemoresistance, we inhibited P-gp with the third-generation inhibitor Tariquidar (TQ). A significant decrease in cisplatin and etoposide sensitivity was seen upon TQ treatment (Fig. 3E, Supplemental Tables 6 and 7). Importantly, TQ did not change the sensitivity of p53 KO or RCP KO to cisplatin or etoposide, suggesting that mutant p53 and RCP promote chemoresistance in a P-gp-dependent manner (Fig. 3E).

Others have shown that beta1 integrin inhibition enhances the sensitivity to chemotherapeutics by preventing activation of EGFR signalling^29^. As we previously demonstrated that mutant p53 promotes beta1 integrin and EGFR signalling, we investigated integrin dependence using Mab16 antibodies that we have shown to inhibit invasion. These antibodies caused a small decrease in cisplatin sensitivity, but no change in etoposide sensitivity (Fig. 3F and Supplemental Table 8), suggesting that integrins might play a small role in cisplatin resistance, but do not play a role in etoposide resistance.

Previously, others have shown that mutant p53 regulates P-gp mRNA expression^30^. We therefore determined P-gp mRNA and protein levels in A431 cells and HCT116 cells, but could not detect significant differences in P-gp mRNA expression upon loss of mutant p53 or RCP expression in either cell line (Fig. 3C, D and Supplemental Fig. 3A–C). Together, these data suggest that an interaction between RCP and P-gp in A431 and HCT116 cells plays a role mutant p53-mediated chemoresistance.

### Mutant p53 promotes RCP and P-gp co-localisation at the plasma membrane in response to cisplatin

**Fig. 4:**
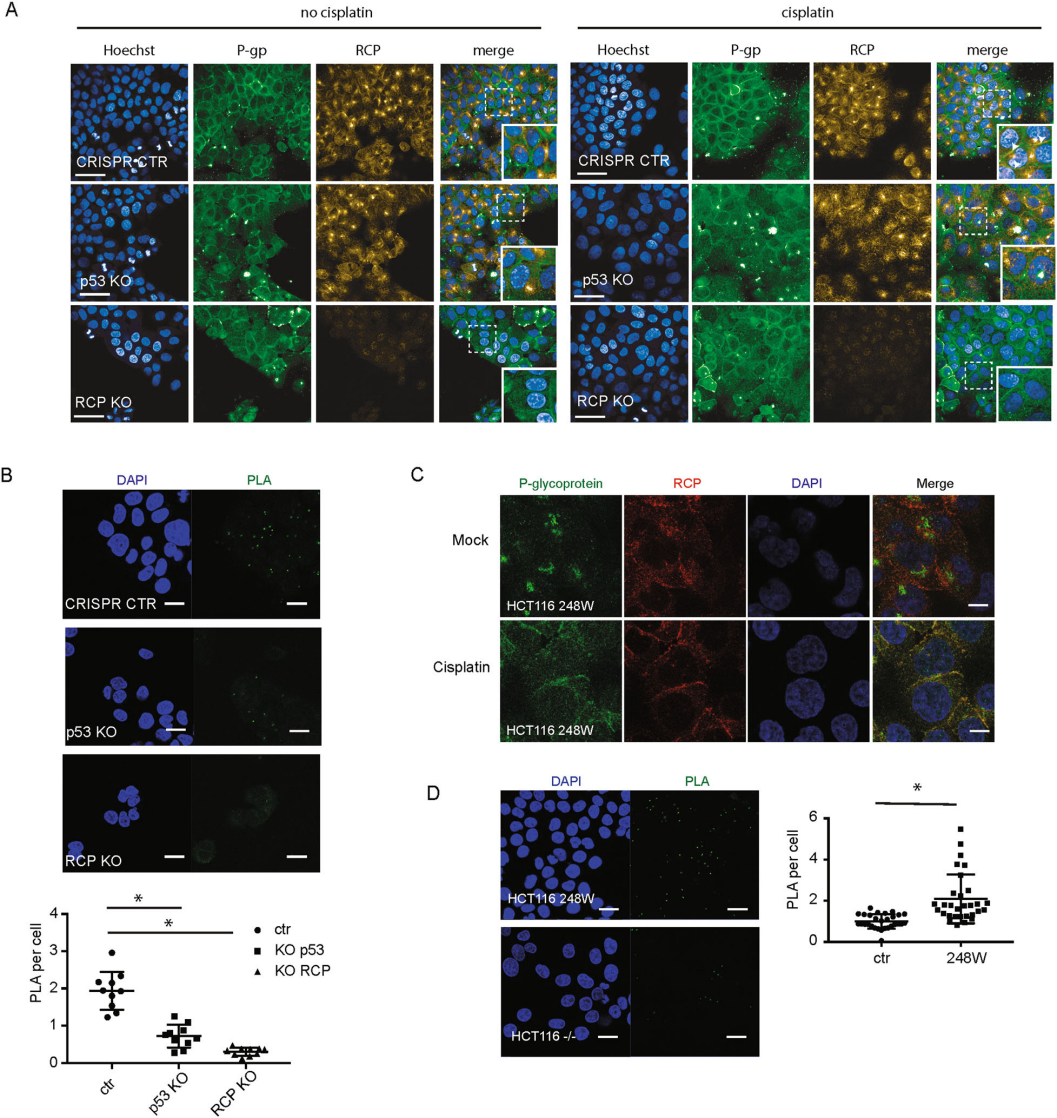
Mutant p53 promotes RCP and P-gp co-localisation. **A** A431 ctr1, A431 RCP KO and A431 p53 KO cells were grown on coverslips and treated with cisplatin 6.7 µM for 2 h. Localisation of endogenous P-gp and RCP was determined with confocal microscopy using antibodies specific for P-gp (Invitrogen Ab, green) and RCP (red). Scale bars are 20 µm. **B** Proximity ligation assays (PLA) in A431 CRISPR control, RCP KO and p53 KO. Images were taken by confocal microscopy (top). Scale bars are 20 µm. The PLA signal was quantified as dots/nucleus for all the cells and plotted in a box and whiskers plot (bottom). Statistical differences were measured using a one-way ANOVA, multiple testing corrected *P* < 0.00001 for both, two-sided. Error bars indicate SD of average values of three independent experiments (three repeats per experiment). **C** HCT116 248W cells were grown on coverslips and treated with cisplatin 6.7 µM for 2 h. Localisation of endogenous P-gp and RCP was determined with confocal microscopy using antibodies specific for P-gp (Santa Cruz Ab, green) and RCP (red). **D** Images (left) and quantification (right) of PLA in HCT116 null and 248W cells with the antibodies specific against P-gp and RCP. Statistical differences were measured using a *t*-test, *P* < 0.00001, two-sided. Error bars indicate SD of average values of three independent experiments (three repeats per experiment).

As RCP controls the trafficking of integrins and receptor tyrosine kinases, we determined the cellular localisation of P-gp in the presence or absence of cisplatin. Antibodies against endogenous proteins revealed a strong perinuclear staining for both P-gp and RCP in A431 cells (Supplemental Fig. 4A). Upon cisplatin treatment a substantial proportion of P-gp and RCP relocalised to the plasma membrane (Supplemental Fig. 4A). Importantly, redistribution of P-gp to the plasma membrane was strongly diminished following RCP or P-gp knockdown. (Supplemental Fig. 4A). As the perinuclear part of the staining for P-gp appeared to be none-specific since it persisted upon P-gp silencing (Supplemental Fig. 4A bottom panels), we also used another antibody. This antibody did not show perinuclear staining, but showed a stronger membrane staining in general, especially upon cisplatin incubation, and was reduced upon P-gp silencing (Fig. 4A and Supplemental Fig. 4B). We used this antibody to determine P-gp localisation in p53 KO and RCP KO cells and confirmed that P-gp relocalises to the plasma membrane in response to cisplatin in mutant p53 cells, but not in p53 KO or RCP KO cells (Fig. 4A). Interestingly, RCP overexpression did not lead to increased membrane localisation of P-gp, suggesting that cisplatin is not increasing RCP expression, but changes RCP function (Supplemental Fig. 4C).

The close proximity of endogenous P-gp and RCP in A431 cells was examined using proximity ligation assays (PLA) (Fig. 4B). Specificity of the signal was determined using single antibodies (supplemental Fig. 4D) and using RCP KO cells (Fig. 4B). Loss of p53 in A431 cells strongly reduced the PLA signal, suggesting that in the absence of p53, less RCP binds to P-gp. A specific increased membrane co-localisation between RCP and P-gp was also observed in HCT116 248W mutant p53 cells upon cisplatin treatment (Fig. 4C and Supplemental Fig. 5A) and a reduced PLA signal was observed in p53-null HCT116 -/- cells, compared to mutant p53 248W cells (Fig. 4D). We also stained MDA MB231 cells for P-gp, but could not detect a clear membrane signal. As MDA-MB-231 cells are more elongated and form less cell–cell interactions, it is likely the signal is too weak to detect on the membrane. However, in response to cisplatin, intracellular vesicles are more easily detected in these cells, with a more pronounced co-localisation of RCP and P-gp in peripheral vesicles (Supplemental Fig. 5B).

**Fig. 5:**
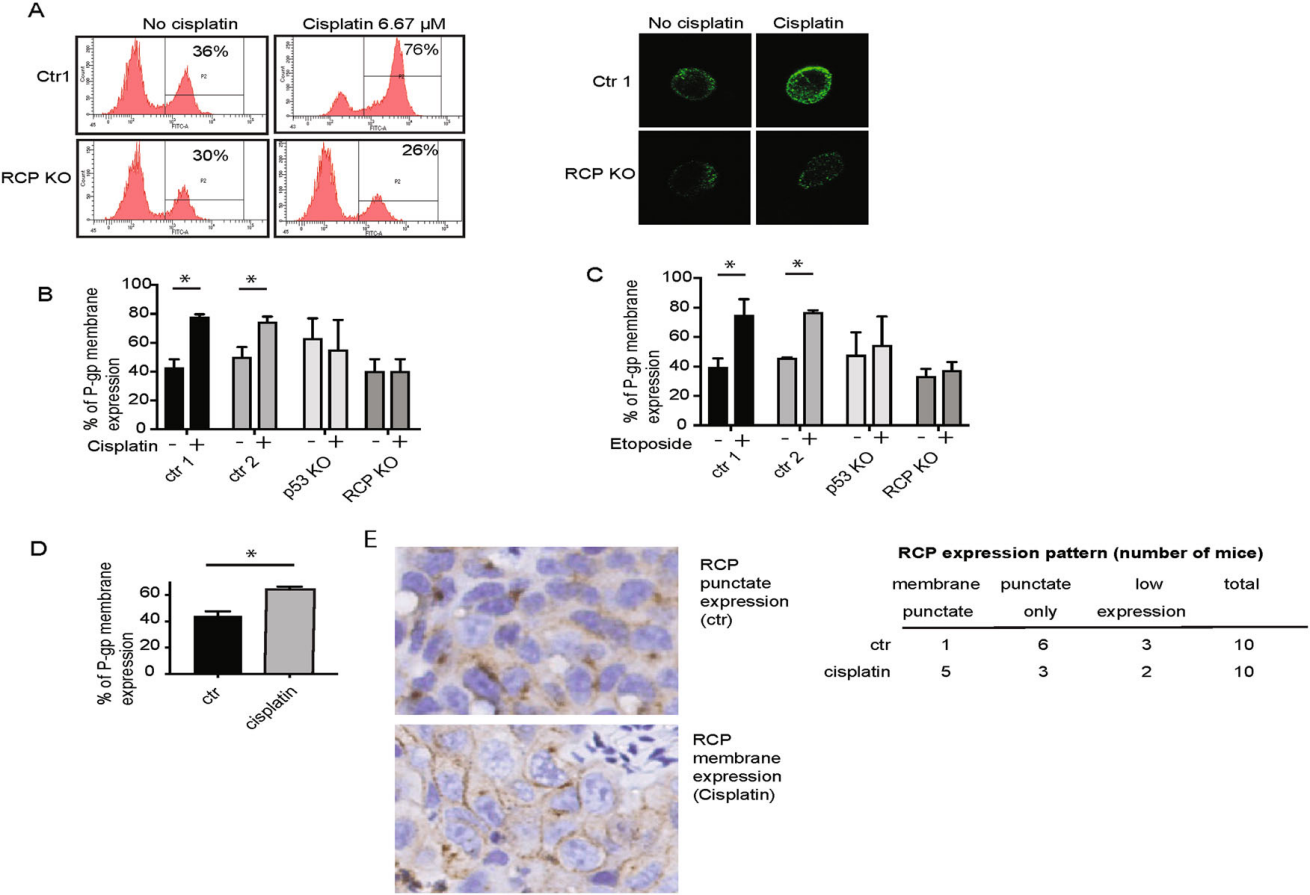
Plasma membrane expression of P-gp is enhanced in mutant p53 cells upon cisplatin treatment. **A** P-gp plasma membrane expression was evaluated by flow cytometry of the A431 control and RCP KO cells treated with cisplatin (6.7 µM for 2 h) (left). Fluorescent images of individual cells are shown on the right, taken after flow cytometry. **B** Quantification of P-gp plasma membrane expression in A431 ctr1, ctr2, RCP KO and p53 KO cells upon cisplatin treatment (6.7 µM for 2 h). Statistical differences were measured using a one-way ANOVA, multiple testing ctr1 ± cisplatin *P* = 0.0001, ctr2 ± cisplatin *P* = 0.0108, two-sided. Error bars indicate SD of average values of three independent experiments. **C** Quantification of P-gp plasma membrane expression in A431 ctr1, ctr2, RCP KO and p53 KO cells upon etoposide treatment (3.4 µM for 2 h). Statistical differences were measured using a one-way ANOVA, multiple testing ctr1 ± etoposide *P* < 0.0001, ctr2 ± etoposide *P* = 0.0002. Error bars indicate SD of average values of three independent experiments. **D** P-gp plasma membrane expression in HCT116 p53 248W/- in the presence and absence of cisplatin (6.7 µM for 2 h). Statistical differences were measured using a *t*-test, two-sided *P* = 0.0011. Error bars indicate SD of average values of three independent experiments. **E** RCP staining in A431 control xenografts from mice exposed to cisplatin or a vehicle control. Numbers of mice that displayed a membrane staining (including punctate staining), punctate staining only, or low RCP expression are indicated on the right.

To quantify plasma membrane levels of P-gp, we used flow cytometry using an antibody that recognises the extracellular domain of P-gp. Specificity was determined using P-gp siRNA (Supplemental Fig. 6A). Cisplatin or etoposide treatment of mutant p53-expressing cells significantly increased the amount of P-gp on the plasma membrane. This increase was not seen in RCP KO or p53 KO cells upon drug treatment (Fig. 5A–C and Supplemental Fig. 6B). Notably, a higher, more variable signal was observed in p53 KO cells, but the signal did not further increase in response to drug treatment. These cells are in general a marginally bigger in appearance and more irregular, which might explain the slightly higher signal.

Consistent with the findings in A431 cells, a cisplatin-mediated increase of P-gp at the plasma membrane was also seen in HCT116 248W cells (Fig. 5D). To prove that plasma membrane expression upon cisplatin treatment underlies the mechanism of cisplatin resistance in vivo, we attempted to stain tumours for P-gp and RCP. Unfortunately, P-gp staining was very low and we could not make consistent conclusions, but RCP was readily detected. Interestingly, as observed in cells, cisplatin treatment lead to an increased membrane staining of RCP in tumours of 5 out of 10 mice, which was only observed in 1 out of 10 control treated tumours (Fig. 5E). These data suggest that P-gp and RCP relocate to the plasma membrane in an RCP-dependent manner in response to chemotherapeutics.

Recently, it was found that RCP can be phosphorylated in response to HGF (Hepatocyte Growth Factor)^31^. Cisplatin has been shown to increase tyrosine phosphorylation of several proteins^32,33^. We therefore investigated if RCP gets phosphorylated in response to cisplatin. We used RCP pulldown and examined the presence of p-tyrosine at the height of RCP in western blots in A431 ctr and p53 KO cells. We could not detect any increase in RCP phosphorylation in any of the conditions (Supplemental Fig. 6C). These data suggest that RCP is not tyrosine phosphorylated in response to etoposide or cisplatin, but do not rule out other RCP phosphorylations in response to chemotherapeutics.

### Mutant p53-induced P-gp membrane expression promotes drug efflux

**Fig. 6:**
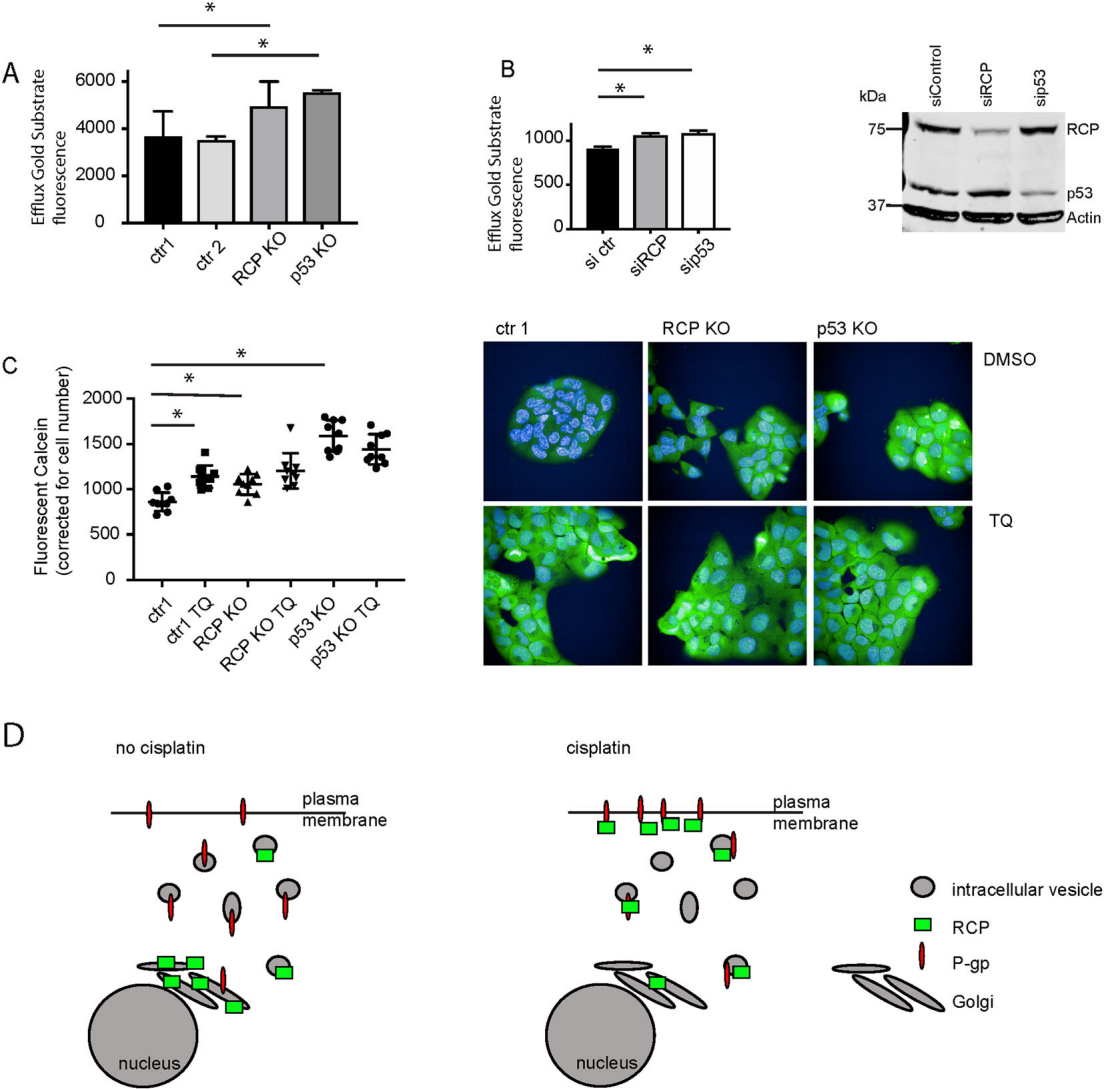
Mutant p53 promotes drug efflux by P-gp. **A** P-gp export function was determined by EFFLUX-ID Gold in A431 ctr1, ctr2, RCP KO and p53 KO cells. Statistical differences were measured using a paired *t*-test. ctr1 vs RCP KO, *P* = 0.0218, ctr2 vs p53 KO, *P* = 0.006, two-sided. Error bars indicate SD of three independent experiments. **B** HCT116 248W/- cells were transfected with 150 pmol of ctr siRNA, siRCP or sip53 for 48 h, followed by EFLUXX-ID Gold assay. Knockdown was confirmed using western blot to detect RCP and p53. Actin was used as loading control. Statistical differences were measured using a one-way ANOVA, sictr vs siRCP *P* = 0.0025, sictr vs sip53 *P* = 0.0009, two-sided. Error bars indicate SD of average values of three independent experiments. **C** Box and whiskers plot showing the calcein AM retention in A431 ctr1, RCP KO and p53 KO cells in response to 0.5 μM Tariquidar (TQ). Individual values of three experiments (three technical repeats) are indicated with error bars representing SD. Statistical differences were measured using one-way ANOVA with multiple testing (two-sided). *P*-values are indicated in Supplemental Table 9 for all conditions. Example images of fluorescent calcein are shown on the right. **D** Schematic model of RCP function in mutant p53 cells in response to cisplatin. In the absence of cisplatin RCP resides in the endocytic recycling complex (ERC) and P-gp is dispersed over various intracellular vesicles. In response to cisplatin, RCP and P-gp relocalised to the plasma membrane in an RCP-dependent manner to facilitate drug efflux.

To determine whether membrane-associated P-gp facilitated drug efflux, we used two different efflux assays^34^, EFFLUX Gold and Calcein. EFFLUX-ID Gold substrate accumulation was measured after 1 h exposure in control, p53 KO or RCP KO A431 cells. Loss of either RCP or mutant p53 caused significant increases in the intracellular retention of this substrate (Fig. 6A). Similar results were found using HCT116 248W cells that were transfected with siRNA to reduce RCP or p53 expression levels (Fig. 6B), although the absolute amount of substrate retained was lower.

To determine P-gp specificity, we used a different efflux assay as the readout is semi-automated microscopy. We used calcein AM and pre-treated cells with TQ to inhibit P-gp function. Calcein levels were higher in RCP KO and p53 KO cells than in control cells (Fig. 6C and Supplemental Table 10). TQ significantly increased calcein levels in ctr cells to the levels seen in RCP KO cells, but did not significantly increase the levels in RCP KO cells (Fig. 6C). The highest levels were observed in p53 KO cells and TQ did not seem to affect calcein levels in these cells. These data support a role for both mutant p53 and RCP in promoting delivery of P-gp to the plasma membrane to facilitate drug efflux.

## Discussion

We discovered that RCP interacts with P-gp and promotes chemoresistance in mutant p53 cells. Our data support a model in which P-gp is localised to recycling endosomes under normal conditions. However, in response to chemotherapeutics, P-gp relocalises to the plasma membrane together with RCP to facilitate P-gp efflux in mutant p53-expressing cells (Fig. 6D).

Previous studies have shown that P-gp is downregulated by wild-type p53 and upregulated by mutant p53^30,35–37^. Our A431 and HCT116 cells did not show a change in P-gp expression on mRNA or on protein levels between p53-null and mutant p53 cells. Our results therefore illustrate that the changes in P-gp resistance to cisplatin or etoposide were not a result of changes in P-gp expression levels in these cells. However, in a tumour, increased P-gp recycling might work in conjunction with increased P-gp expression and so further enhance chemoresistance. Some reports suggested a correlation between mutant p53 and P-gp expression in human cancers, but others did not find a correlation^38–41^. Our work could suggest that in cancers in which a direct correlation in expression is not found, mutant p53 expression might still be correlated with P-gp plasma membrane expression and drug efflux.

Only a limited number of studies have investigated the localisation of P-gp in response to drugs^22^. Many chemotherapeutics have been found to be substrates for P-gp, including etoposide^42^. However, cisplatin is not a direct substrate for P-gp, but an increased expression of P-gp in cisplatin-resistant cell lines or a sensitization to cisplatin upon downregulation of P-gp has been observed^43,44^. DeMeule et al. furthermore detected an increase in the expression of P-gp at the plasma membrane in vivo in rat kidneys in response to cisplatin^45^. P-gp-mediated cisplatin resistance in mutant p53 cells is what we see in our cell lines. Interestingly, this resistance coincided with cisplatin-driven translocation of P-gp to the plasma membrane that was dependent on RCP and mutant p53. This raises the question—why does cisplatin drive P-gp to the plasma membrane, if cisplatin is not directly exported by P-gp? P-gp has been shown to inhibit apoptosis, induced by a variety of factors that are not P-gp substrates, including ultrasound^46^. Although the mechanism is not fully understood, it is proposed to be linked to apoptosis-related endogenous substrate efflux. The main endogenous substrates of P-gp are thought to be oxidised lipids. Reactive oxygen species are elevated following cisplatin addition and cisplatin has been shown to change the lipid composition of the plasma membrane^47,48^. Our results, therefore, point towards a role for RCP in delivering P-gp to the plasma membrane to mitigate toxicity in response to cisplatin. Our study also reveals that etoposide, which is a P-gp substrate, results in RCP-dependent delivery of functional P-gp to the plasma membrane. In addition, two artificial substrates of P-gp were retained in cells that had lost RCP or mutant p53 expression. These data strongly point to a role for RCP recycling of P-gp in chemoresistance.

A remarkable finding in our study is the fact that RCP KO cells did not form tumours as quickly as control cells and p53 KO cells did not form tumours in xenograft studies. This was not expected given that loss of RCP did not impair tumour growth in other studies^31^ and loss of p53 is widely used to generate tumours. Perhaps these differences can be attributed to the fact that we knocked out p53 or RCP in cells that had already acquired a gain-of-function mutation in p53 R273H. Experiments in mice and cell lines have revealed that cancer cells can become dependent on p53 mutations and that a loss of mutant p53 can impair tumour growth and cell survival^49,50^. This is in line with the observation that p53 KO and RCP KO cells were impaired in anchorage-independent growth. Integrins are involved in the attachment of cells to their matrix and our previous work detected a decrease in the recycling of the alpha5/beta1 integrin to the cell surface in A431 cells that had lost RCP^20^.These data could therefore suggest that loss of RCP or p53 in mutant p53 cells impairs anchoring and settling of the xenografted tumour cells.

Our data suggest that RCP-dependent endosomal trafficking can regulate the function of several transmembrane proteins and thereby impact on the invasion, metastasis and chemoresistance of mutant p53-expressing tumour cells. Beta1 integrin has been shown to promote EGFR recycling and signalling, which led to increased sensitivity to cisplatin in A549 cells^29^. We inhibited integrin recycling using the Mab16 antibody and noticed a small increase in sensitivity to cisplatin, but not to etoposide. These data could indicate that cisplatin resistance is partly driven in an integrin-dependent manner. It is interesting that there is a difference between etoposide and cisplatin, which warrants further investigation into the details of the RCP interaction with EGFR, integrin and P-gp in response to different chemotherapeutics. We also do not exclude the possibility that other transporters are regulated by RCP in mutant p53 cells. We validated P-gp specificity using siRNA targeting P-gp or using TQ, suggesting that the effects we saw were specific for P-gp. However, other transporters might contribute as well or regulate resistance to other chemotherapeutics. Other less well-known ABC transporters were detected in our mass-spectrometry pulldown experiments and warrant further investigation.

It will also be important to determine how RCP is regulated by chemotherapeutics. More recently, RCP was shown to be phosphorylated by LMTK3 to promote cell–cell repulsion in response to the growth factor HGF by regulating EphA2 localisation^31^. We looked at RCP tyrosine phosphorylation as cisplatin has been shown to increase tyrosine phosphorylation EGFR in cancer cells^51^, but our p-tyrosine pulldowns did not show any tyrosine phosphorylation on RCP. Cisplatin has also been shown to regulate serine phosphorylation of EGFR, leading to internalisation of EGFR, which was independent of tyrosine phosphorylation^52^. Whether or not RCP is phosphorylated upon chemotherapeutic exposure remains to be elucidated.

To our knowledge this is the first time that RCP is implicated in chemoresistance in cancer cells. Given that RCP is a major hub in the regulation of cell invasion and metastasis, inhibition of RCP could be interesting for therapeutic intervention in mutant p53 cancer cells.

## Materials and methods

### Cell lines and culture conditions

H1299, MDA-MB-231 and A431 were obtained from ATCC (www.ATCC.org). Stable H1299 273H or empty vector cells were described before^20^. HCT116 cells were a gift from Dr. Vogelstein. All cells were cultured in Dulbecco’s Modified Eagle’s medium (DMEM) (Thermo Fisher Scientific), supplemented with 10% FBS (Thermo Fisher Scientific) and 5% pen/strep (Thermo Fisher Scientific) at 37 °C and 5% carbon dioxide. Cells were tested for mycoplasma and authenticated. Stable clones of H1299 were generated by transfecting the cell lines using Genejuice (Merck Millipore) according to manufacturer’s instruction followed by selection with 600 µg/mL G-418 (Sigma).

### Cloning

A web-based CRISPR design tool (http://crispr.mit.edu) was used for making RCP CRISPR constructs. The following CRISPR guides against RCP were used: fw- *CACC*GAAGTACGCCACCTCCGTGT, rev - *CAAA*ACACGGAGGTGGCGTACTTC. Then, 1 µg of pX330 plasmid (Addgene) was digested with 1 U/µL of FastDigest BbsI (Thermo Scientific) restriction enzyme at 37 °C for 30 min in the presence of 1 U/µL Fast alkaline phosphatase (Thermo Scientific). The digested plasmid was agarose gel purified (QIAquick Gel extraction kit, Qiagen). Further, each pair of oligos (100 pmol) were phosphorylated and annealed in presence of 500 U/µL of T4 polynucleotide kinase at 37 °C for 30 min and 95 °C for 5 min and then ramped down to 25 °C at 5 °C/min to form oligo duplexes. This was followed by a ligation reaction with digested pX330 plasmid, oligo duplex (1:250) and quick ligase (New England Biolabs) along with 2X quick ligation buffer for 10 min at RT. The ligated CRISPR cas9 construct along with RCP specific sequence was transformed in a competent bacteria DH5alpha (NEB).

### CRISPR/Cas9-mediated knockouts

The generation of CRISPR KO lines was described by Mackay et al.^53^; 10 µg of the above-generated CRISPR Cas9 RCP construct or pre-made GFP CRISPR constructs (Sigma, USA) against p53 (TCCATTGCTTGGGACGGCAAGG) or (GACTGCTTGTAGATGGCCA) empty vector controls (CRISPR ctr1 and ctr2) were transfected in A431 cells using lipofectamine^®^ 2000 Transfection Reagent (Thermo scientific) and Opti-MEM™ I Reduced Serum Medium (Thermo scientific). p53 KO fluorescent cells were selected by FACS sorting (BD FACS Aria).

RCP KO cell were selected with 5 µg/mL Puromycin (Sigma). The cells resistant to the drugs or the sorted cells were seeded sparsely and clonal colonies were picked up and verified for their loss of RCP or p53 expression by SDS-PAGE. Notably, p53 KO A431 cells were cultured in the DMEM medium supplemented with hydrocortisone (0.5 mg/mL), cholera toxin (100 ng/mL, Sigma), insulin (10 µg/mL, Sigma) and EGF (20 ng/mL, Sigma) until clones were confirmed.

### siRNA and transfection procedures

Table 1 lists the used siRNAs. Cells were transfected in a 6-well dish with 150 pmol ctr, RCP and p53 siRNA or 250 pmol P-gp sRNA along with 6 μl lipofectamine (or 10 μl for P-gp siRNA) Lipofectamine^®^ 2000 and Opti-MEM™ (Thermo Fisher Scientific) I Reduced Serum Medium for 72 h. For p53 knockdown, only one siRNA was used that we have thoroughly investigated for specificity on all p53 isoforms and that does not interfere in p63 or p73 expression. For RCP and P-gp knockdown, a cocktail of 4 siRNAs was used.

**Table 1:** siRNA.

siRNA	Sequence	Manufacturer
control siRNA	Dharmacon D-001810-10-20	Dharmacon
siRCP_1	AAACAGAAGGAAACGAUAA	Eurofins
siRCP_2	GGAAAGAUGUAAAUCAGCA	Eurofins
siRCP_3	AGUGAGAACUUGAACAAUG	Eurofins
siRCP_4	CCACCAAGGUUGCUAACUG	Eurofins
si P-gp_1	GCAGGAAAUUUAGAAGAUC	Eurofins
si P-gp_2	UGAGGAUGUUCUGUUAGUA	Eurofins
si P-gp_3	UUUUCAUGCUAUAAUGCGA	Eurofins
si P-gp_4	CGUUUGUCUACAGUUCGUA	Eurofins
si p53	GACUCCAGUGGUAAUCUAC	Eurofins

### Cell viability tests

Cell viability was determined by using MTT cell proliferation kit (R&D systems) on a Tecan Infinite m200 pro reader at 570 nm absorbance according to the manufacturer’s instructions. In all, 10,000 cells were plated (48 h after siRNA transfection) in 96-well plates and incubated in cisplatin or etoposide for 72 h. Measurements were done as four technical repeats in three independent experiments. Resazurin survival assays were used to have a larger dynamic range and were measured at 575 nm in the fluorescent channel of a Spectramax MS5 spectrophotometer (Excitation at 555 nm). Measurements were done as three technical repeats in three independent experiments. For this assay, 20,000 cells were cultured per well of 96 wells tissue culture dishes, and incubated with cisplatin or etoposide for 72 h. GFP RCP and GFP cells were selected using FACS sorting and plated 48 h after sorting.

### Western blot analysis

Western blot was described previously^21^. In short, protein lysates were prepared by washing the cells twice in ice-cold PBS and lysed on ice with RIPA buffer (1% Ipegal, 1% sodium deoxycholate, 0.1% SDS, 0.15 M NaCl, 0.01 M sodium phosphate (pH 7.2)) and supplemented with complete protease inhibitor tablet (Roche). Lysates were run on SDS-PAGE and blotted on nitrocellulose. The blots were probed with the following primary antibodies: P-gp ((clone G-1), 1:250, Santa Cruz Biotechnology), Rab-coupling protein (1:1000, D9D8P, Cell Signalling), p53 (DO-1) (1:1000, Santa Cruz Biotechnology) and actin (1:5000,C2, Millipore). Secondary antibodies were donkey-anti-mouse 800 or donkey-anti-rabbit 680 and 800 (LI-COR Biotechnology) using the Li-Cor Odyssey, and anti-mouse HRP conjugated (1:5000, Santa Cruz Biotechnology). Blots for P-gp expression were developed with SuperSignal™ Western Blot Enhancer (Thermo Scientific).

### Immunoprecipitation

GFP IPs were described before^21^. For RCP/ P-gp IPs, cells from each 10 cm tissue culture dish were lysed by scrapping in 200 µL of NDLB (5 M NaCl, 1 M Tris-HCl, pH 7.5, 0.5 M EDTA, 0.5 M EGTA, 0.5 M NaF, 0.1 M NaVO4) and supplemented with complete protease inhibitor tablet, 0.15% Tween 20 and 10% SIGMAFAST™ Protease Inhibitor Cocktail Tablet (Sigma). Scraped cells were passed through a 27.5 G needle (5X) and centrifuged at maximum speed for 10 min at 4 °C. For each protein lysate, 40 µL of the sheep-anti-mouse IgG dynabeads (Thermo scientific) or 40 µL of the Dynabeads™ Protein G (Thermo scientific) were coupled with antibodies against P-gp (8 µg), GFP (0.75 µg, Ab6556, Abcam) or RCP (4 µL, D9D8P, Cell Signalling) for 1 h at 4 °C and antibody coupled beads were further incubated with the protein lysates overnight at 4 °C under constant rotation. Unbound proteins were washed by 4X washing with the NDLB lysis buffer and eluted in 40 µL of 2X reducing sample buffer and boiled for 10 min. One example is shown, but verified independently in two additional repeats.

### Immunofluorescence, proximity ligation assays and confocal imaging

Immunofluorescence was described previously^53^. Cells were grown on the glass cover slips in 24 wells tissue culture plate until reaching 70% confluency, washed with ice-cold PBS and fixed with 4% paraformaldehyde (Thermo scientific) for 15 min at 4 °C. Cells were washed with PBS and permeabilised with 0.1% triton-X for 10 min at RT, blocked in 5% BSA (Sigma) at RT for 1 h, followed by ON first Ab incubation in block buffer at 4 °C, and 1 h of secondary Ab. Antibodies were used in the following dilutions: RCP (D9D8P, Cell Signalling 1:75) and P-gp (SC 31313 1:50, Invitrogen P-gp F4 1:50). Images of one of the three independent experiments are shown.

For PLA, P-gp (SC 31313) and RCP (D9D8P, Cell Signalling) were used in the same concentrations as above in combination with mouse and rabbit plus and minus probes, according to the manufacturers’ protocol (Duolink, Sigma). Images were taken with an inverted confocal fluorescent microscope (Zeiss). For quantification, >30 fields (6 images per 2 repeats in three independent experiments) were quantified using ImageJ to calculate number of dots per nuclei. For quantitative CC3 staining, 96-well plates were imaged and analysed for the CC3 (1:300, Asp175, Cell Signalling) and nuclear staining (DAPI, Thermo Fisher Scientific) in a Cellomics ArrayScan VTI with a HCS Studio 6.6.0 software (Thermo scientific). Images were taken of duplicate samples in three independent experiments.

### Clonogenic assay and anchorage-independent growth assays

A431 cells were grown for 24 h on 6-well tissue culture dishes and treated with cisplatin (Santa Cruz Biotechnology) or etoposide (Sigma). After 24 h of treatment, 1000 cells were seeded per well. Once visible colonies were formed, colonies were fixed with 4% PFA for 10 min at 4 °C and stained with 0.01% crystal violet in distilled water for 30–45 min. Excess of crystal violet was washed with water and allowed to dry. Plates were scanned in the Li-Cor Odyssey (700 nm) and colony numbers were analysed using ImageJ (https://imagej.nih.gov/ij/).

For anchorage-independent growth, 1000 A431 cells were grown in soft Agar (5%) for 20 days. Colonies were imaged using bright-field microscopy and colony number and size were determined using ImageJ in three independent experiments (two technical repeats, 3 images per condition containing >100 colonies each).

### EdU staining

A431 CT, RCP KO and p53 KO cells were incubated with 10 µM EdU for 1 h, fixed, incubated with the Click-iT^®^ (Click-iT™ EdU (5-ethynyl- 2′-deoxyuridine) Alexa Fluor™ 488 Flow Cytometry Assay Kit, Life Technologies) reaction cocktail (30 min, 20 °C), permeabilised with saponin and counterstained with Hoechst 33342. Cells were imaged using the PerkinElmer Opera Phenix confocal platform. The proportion of green nuclei (EdU) to blue nuclei (Hoechst 33342) was determined in 4 fields, each containing 350 cells on average.

### P-gp cell surface expression

Cells were treated with cisplatin (6.7 µM) or etoposide (3.4 µM) for 2 h. Cells were trypsinised, washed 3x with ice-cold PBS and fixed with 4% PFA for 15 min at 4 °C. Cells were then washed 3x with PBS, blocked in 5% BSA-PBS at RT for 1 h and incubated in P-gp (1:50) (ProteinTech, 223361) in 1% BSA-PBS overnight at 4 °C. Cells were then washed 3x with cold PBS and incubated with secondary antibody (AlexaFluor alpha rabbit 594 1:250, Life Technologies) in 1% BSA-PBS for 1 h at RT. After 3 PBS washes, P-gp expression was measured by flow cytometry in three independent experiments.

### Efflux assays

P-gp substrate accumulation was determined using EFLUXX-ID^®^ Gold multidrug resistance assay kit (Enzo Life Sciences, Inc.) according to the manufacturers’ protocol and measured by flow cytometry. For Calcein AM retention, some cells were pre-treated with 0.5 μM TQ for 24 h. Calcein AM was added to a final concentration of 0.01 μM and cells were incubated for 20 min at 37 °C in the incubator. Cells were washed with pre-warmed medium and incubated for 10 min at 37 °C to ensure optimal retention and 5 μg/mL Hoechst 33342 was added to stain nuclei in this incubation time. Cells were imaged on an Opera Phenix high-content screening microscope. The proprietary software Harmony was used to find the nuclei (Hoechst) and cell outline of calcein and fluorescent intensity of calcein was determined per cell. Measurements were taken from duplicate wells in three independent experiments.

### Xenografts

Animal experiments were conducted under UK Home Office licence 70/8655. Six groups of ten 40+ day-old balb/c nude/male mice (Jackson laboratories), housed at the Division of Biomedical Services at the University of Leicester were randomly allocated to each group (10 A431 control, 10 A431 control + cisplatin, 10 A431 RCP KO, 10 A431 RCP KO + cisplatin) to avoid bias in age and litter of mice and housed as 5 mice per group with food and water ad libitum. Numbers per group were based on the variation seen in a pilot experiment of A431 control cells. Mice were randomly numbered and all subsequent measures were done blinded. One mouse was low in weight (<20 g) in the RCP KO group on the day of cell injections and was therefore excluded from the study. Another mouse in the RCP KO group was excluded due to a significant leakage of cells and no tumour was detected at the end of the study. For xenograft experiments, 100 µL cell solution containing 10,000 cells and Matrigel (1:1, PBS) was injected subcutaneously in mice anaesthetised with 1.5% isoflurane. Tumours were grown for 5 days after which cisplatin was administered intraperitoneally at 5 mg/kg, which was repeated 5 days later. Whole-body fluorescent images (IVIS) were taken in a blinded manner 12 and 14 days after xenograft injections, and mice were sacrificed after the last images were taken when the first tumour reached our licence maximum stipulated size of 1200 mm^3^ as measured by calliper measurements. The spectral unmixing tool was used to separate the fluorophore signal from background signal in all mice. Prior to imaging, mice were anaesthetised using 5% isoflurane in oxygen and maintained on 2% isoflurane for imaging. Animals were placed in the camera chamber with sterile eye lubricant. Mice were sacrificed after the last images and tumours were processed for histology. Fluorescence signals were quantified by using Living Image v. 4.5 software (PerkinElmer Inc.)

### mRNA analysis

For A431 cells, the RNeasy Plus Mini Kit (Qiagen) was used to isolate RNA according to the manufacturer’s instructions. cDNA was synthesized using M-MLV Reverse Transcriptase (Promega) with 1 µg RNA in a final volume of 20 µL, according to the manufacturer’s instructions. RT-qPCR was prepared using the Fast SYBR green master mix (Thermo Scientific) according to the manufacturer’s instructions in 15 µL and with 1000 nM of each primer, and run in the QuantStudio 6 Flex Real-Time PCR System (Thermo Scientific). Cycling conditions: denaturation (95 °C, 3 s), annealing (59 °C, 30 s), extension (72 °C, 30 s). Primers used (5′ – 3′): GAPDH fwd: AATCCCATCACCATCTTCCA, GAPDH rev: TGGACTCCACGACGTACTCA, P-gp fwd: CCGAACACATTGGAAGGAA, P-gp rev – CTTTGCCATCAAGCAGCAC.

For HCT116 cells, the Total RNA Purification Kit (Norgen Biotek) was used to isolate RNA according to the manufacturer’s instructions. cDNA was synthesized using the High-Capacity cDNA Reverse Transcription Kit (Applied Biosystems) with 1 µg RNA, in a final volume of 20 µL, according to the manufacturer’s instructions. RT-qPCR was prepared using the PowerUp SYBR Green Master Mix (Applied Biosystems) according to the manufacturer’s instructions in 15 µL and with 500 nM of each primer, and run in the QuantStudio 3 Real-Time PCR System (Thermo Scientific). Cycling conditions: Denaturation (95 °C, 5 s), annealing + extension (60 °C, 20 s). Primers used (5′ – 3′): GAPDH as for A431 cells, P-gp fwd: ACAGAGGGGATGGTCAGTGT, P-gp rev – TCACGGCCATAGCGAATGTT.

### Histology, immunohistochemical staining and image analysis

The tumours were immediately fixed with zinc formalin (Sigma) overnight at 4 °C and paraffin embedded (FFPE block). Then, 5 µm sections were taken from each block and stained with H&E (Sigma) using the Shandon Varistain autostainer. Immunohistochemical staining was performed using a chromogenic DAB method. Assays were performed on an automated staining platform (Ventana Discovery Ultra, Roche Tissue Diagnostics/Agilent Dako Link 48, Agilent) using the conditions depicted in Table 2, and counterstained with Harris haematoxylin.

**Table 2:** Conditions for Immuno Histochemistry.

Platform	Primary antibody	Source	Primary concentration	Retrieval	Primary incubation	Secondary antibody	Secondary incubation	Visualisation system
Ventana	Cleaved caspase 3	Cell Signalling, ASP175	1:300	CC1, pH 9, 32 min, 97 °C	52 min	Omnimap anti-rabbit HRP	12 min	ChromoMap DAB
Dako	p53	Dako, clone DO-7	1:3000	Flex TRS High, pH 9, 20 min	20 min	Flex HRP	20 min	Flex DAB
Leica Bond RX, refine kit	RCP (Rab11FIP1)	Cell Signalling	0.15 µg/mL (1:100)	ER1, pH 6, 20 min, 98 °C	30 min	Flex HRP rabbit	20 min	Flex DAB

For CC3 quantification, stained slides were imaged at 40x magnification with the Hamamatsu Nanozoomer XR digital slide scanner and analysed using Visiopharm, blinded to the researcher. Two analysis protocol packages were developed: first tumour regions were identified using a supervised decision forest classification method trained on the counterstained Immuno Histochemistry image to outline the tumour as a region of interest. Subsequently, CC3-positive tumour areas were identified and the percentage of CC3 positivity was calculated as, CC3 + tumour area/total tumour area. Percentage of CC3 staining was calculated as CC3 + tumour area/total tumour area.

### Graphs and statistics

Graphpad Prism was used to make graphs and to determine statistical differences. Sample size, replicates, repeats and statistical methods are indicated in the legend of each figure. For most experiments, three independent experiments were done with two or three technical repeats depending on the variation in measurement.

## Supplementary information

Supplemental Figure legends.

Supplemental Figure 1.

Supplemental Figure 2.

Supplemental Figure 3.

Supplemental Figure 4.

Supplemental Figure 5.

Supplemental Figure 6.

Supplemental Table 1.

Supplemental Table 2.

Supplemental Table 3.

Supplemental Table 4.

Supplemental Table 5.

Supplemental Table 6.

Supplemental Table 7.

Supplemental Table 8.

Supplemental Table 9.

Supplemental Table 10.

Supplemental Table 11.
