# Supplementary material for: Mutant p53 promotes RCP-dependent chemoresistance coinciding with increased delivery of P-glycoprotein to the plasma membrane
Source: Cell Death Dis. 2021 Feb 24;12(2):207. doi: 10.1038/s41419-021-03497-y (PMC7904762; doi:10.1038/s41419-021-03497-y)
Supplement: Supplementary file 1 — Supplemental Figure legends. [file 41419_2021_3497_MOESM1_ESM.docx]

**Supplemental Figure 1**

(A) Cell survival was determined for HCT116 null and p53 mutant (248W/-) in MTT cell proliferation assay upon treatment with cisplatin or etoposide for 48 hours. Error bars show SD of three independent experiments. Statistical significance was determined using a paired one-way ANOVA adjusted for multiple testing (two-sided), p-values are indicated in supplemental table 10. (B) HCT116 248W/- cells were transfected with RCP siRNA or control siRNA and subjected to cisplatin treatment. Cell survival was determined using an MTT assay. Error bars show SD of three independent experiments. Statistical significance was determined using a paired one-way ANOVA adjusted for multiple testing (two-sided), p-values are indicated in supplemental table 10. Knockdown was verified by western blot for RCP using actin as loading control (right panels). (C) MDA MB231 cells were transfected with RCP siRNA or control siRNA and subjected to cisplatin treatment. Cell survival was determined using an MTT assay. Error bars show SD of three independent experiments. Statistical significance was determined using a paired one-way ANOVA adjusted for multiple testing (two-sided), p-values are indicated in supplemental table 10. Knockdown was verified by western blot for RCP and p53 using actin as loading control (right panels).

**Supplemental Figure 2**

(A) Histological slides with RCP staining in ctr 1 A431 or RCP KO A431. (B) EdU incorporation in A431 control and A431 RCP KO cells as analysed by immune fluorescence and quantified. Average values are shown and error bars indicate SD. (C) Representative brightfield images of anchorage independent colony formation assays of A431 ctr1, ctr2, p53 KO, RCP KO and parental A431 cells. Colony numbers (left) and colony size (right) are quantified from images of three independent experiments Error bars indicate SD of three experiments with three technical repeats (9 measurements are shown in a box and whiskers plot). Statistical analysis was done using a one-way ANOVA corrected for multiple testing (two-sided). P-values are indicated in supplemental table 11 (D) Images of control and cisplatin treated tumours of A431 control of A431 RCP KO cells after dissection. NT= no tumour

**Supplemental Figure 3**

(A) P-gp mRNA expression levels in A431 ctr, A431RCP KO and A431 p53 KO were determined by qRT PCR. Error bars represent SD of three experiments (B) P-gp mRNA expression levels in HCT116 -/- and HCT116 248W cells was determined by qRT PCR. Error bars represent SD of three experiments. (C) Protein expression of RCP, p53 and P-gp using western blot. Actin was used as loading control.

**Supplemental Figure 4**

(A) A431 parental cells were grown on coverslips and transfected with siRNA against RCP or P-gp. Cells were treated with 6.7uM cisplatin for 2 hrs and P-gp and RCP localisation was determined with confocal microscopy using antibodies specific for P-gp (green) and RCP (red). DAPI was used to stain nuclei. Scale bars are 20 µm. (B) A431 ctr cells were transfected with ctr or P-gp siRNA and grown on coverslips. Cells were treated with 6.7uM cisplatin for 2 hrs and P-gp and RCP localisation was determined with confocal microscopy using antibodies specific for P-gp (green) and RCP (red). DAPI was used to stain nuclei. (C) GFP-RCP was expressed in A431 ctr1 cells and p-gp localisation was determine with IF (Invitrogen ab), Dapi was used to stain nuclei, scale bar indicates 10 µm (D) PLA of RCP and P-gp was optimised using both antibodies and each ab alone as indicated in the figure. DAPI was used to stain nuclei. Scale bars are 20 µm

**Supplemental Figure 5**

HCT116 248W cells were grown on coverslips and transfected with siRNA against RCP or P-gp. Cells were treated with 6.7 µM cisplatin for 2 hrs and P-gp (SC) and RCP localisation was determined with confocal microscopy using antibodies specific for P-gp (green) and RCP (red). DAPI was used to stain nuclei. Scale bars are 20 µm. (B) MDA MB231 cells were grown on coverslips and treated with cisplatin 15 µM for 2 hrs. P-gp (SC) and RCP localisation was determined with IF. DAPI was used to stain nuclei

**Supplemental Figure 6**

(A) A431 parental cells were transfected with siRNA against RCP or P-gp and p-gp membrane expression was determined using flow cytometry (bottom). Knockdown was verified by western blot with actin as loading control. (B) FACS images of ctr and p53KO A431 cells treated with cisplatin (1 hr). (C) IP of RCP in A431 ctr1, A431 KO RCP or A431 KO p53 cells, treated with EGF (10 min), cisplatin (1 hr) or etoposide (1hr). Shown are the inputs for p-tyr, RCP and GapDH and the IPs for p-tyr and RCP. A raw image from the Li-Cor is shown to show that no RCP is pulled down in the p-tyr IP.

**Supplemental Table 1**

P-values and IC50 values of Figure 1A cisplatin

**Supplemental Table 2**

P-values and IC50 values of Figure 1A etoposide

**Supplemental Table 3**

P-values and IC50 values of Figure 1B cisplatin

**Supplemental Table 4**

P-values and IC50 values of Figure 1B etoposide

**Supplemental Table 5**

P-values of Figure 1C

**Supplemental Table 6**

P-values and IC50 values of Figure 3E cisplatin

**Supplemental Table 7**

P-values and IC50 values of Figure 3E cisplatin

**Supplemental Table 8**

P-values and IC50 values of Figure 3F

**Supplemental Table 9**

P-values of Figure 6C

**Supplemental Table 10**

P-values of Supplemental Figure 1

**Supplemental Table 11**

P-values of Supplemental Figure 2C
