# Supplementary figures and images for: Mutant p53 promotes RCP-dependent chemoresistance coinciding with increased delivery of P-glycoprotein to the plasma membrane
Source: Cell Death Dis. 2021 Feb 24;12(2):207. doi: 10.1038/s41419-021-03497-y (PMC7904762; doi:10.1038/s41419-021-03497-y)

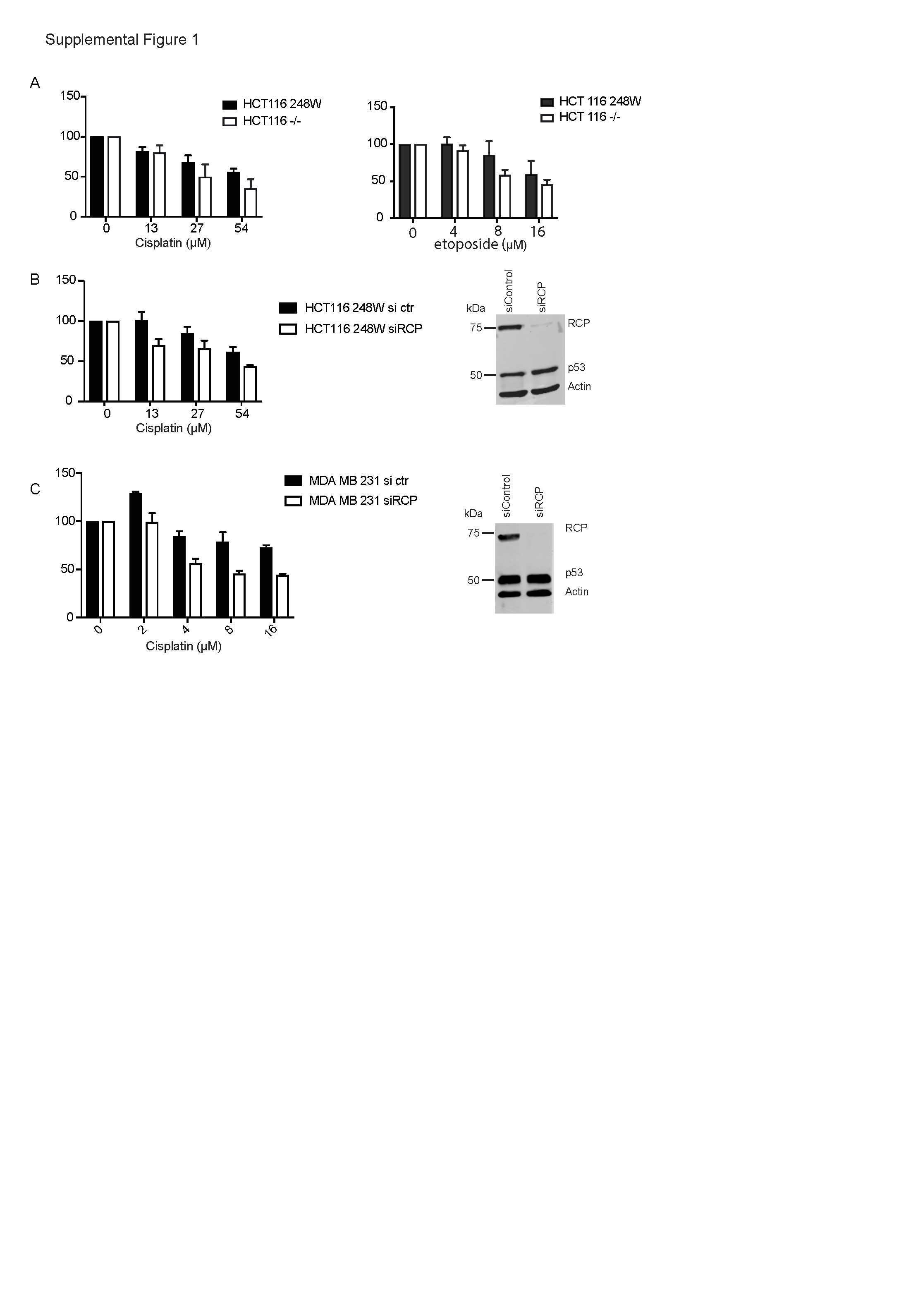

Supplement: Supplementary file 2 — Supplemental Figure 1. [file 41419_2021_3497_MOESM2_ESM.tif]

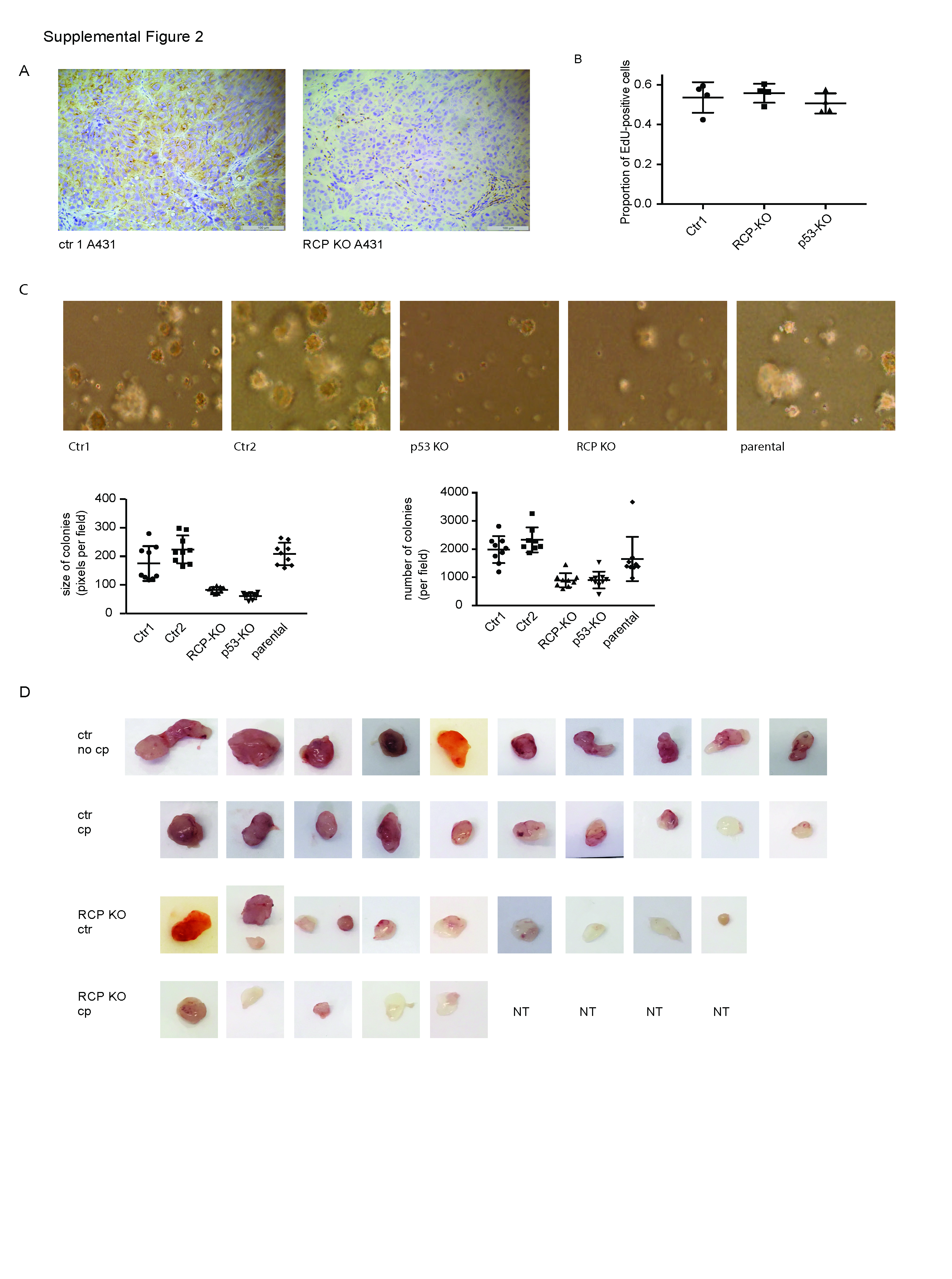

Supplement: Supplementary file 3 — Supplemental Figure 2. [file 41419_2021_3497_MOESM3_ESM.tif]

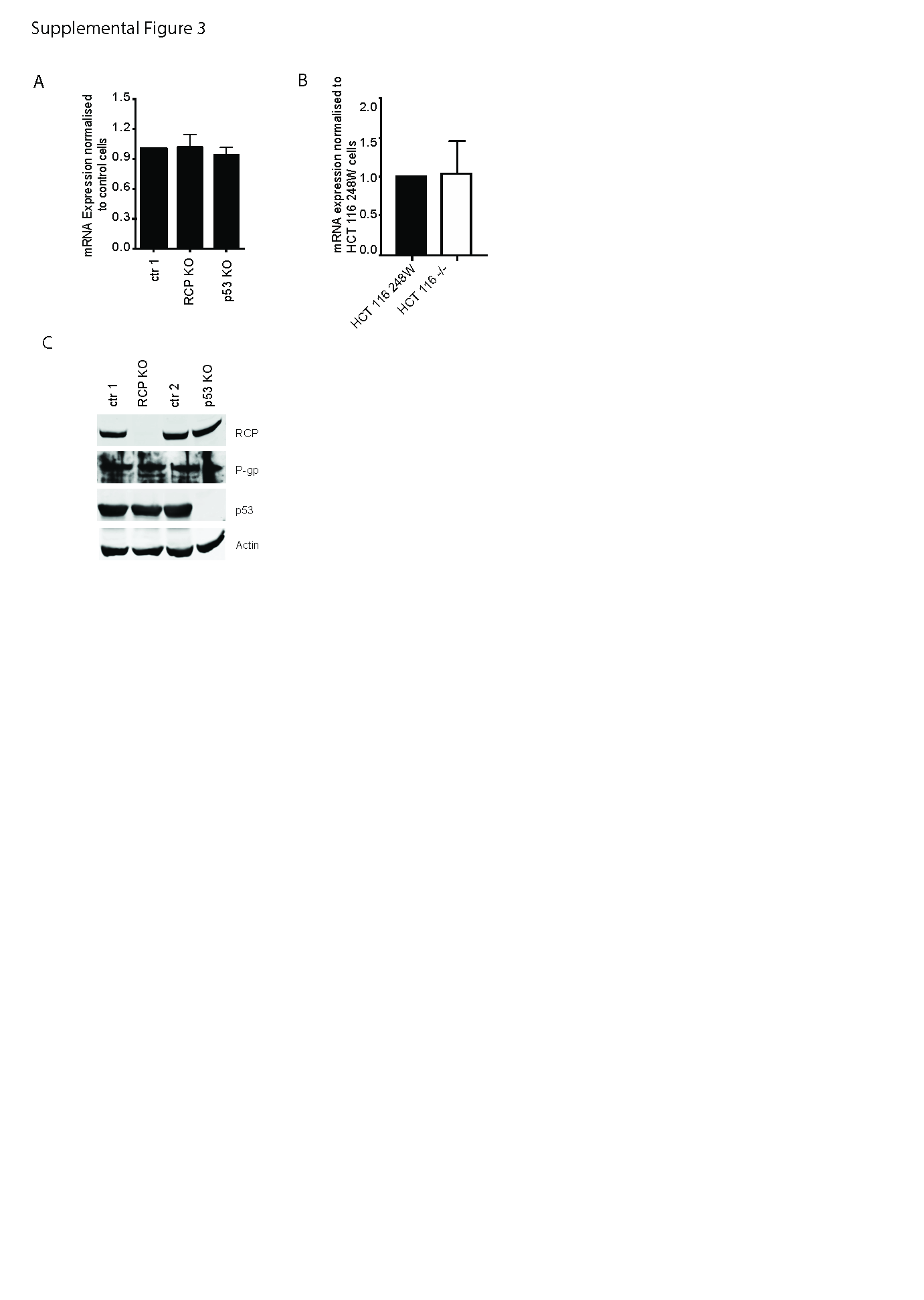

Supplement: Supplementary file 4 — Supplemental Figure 3. [file 41419_2021_3497_MOESM4_ESM.tif]

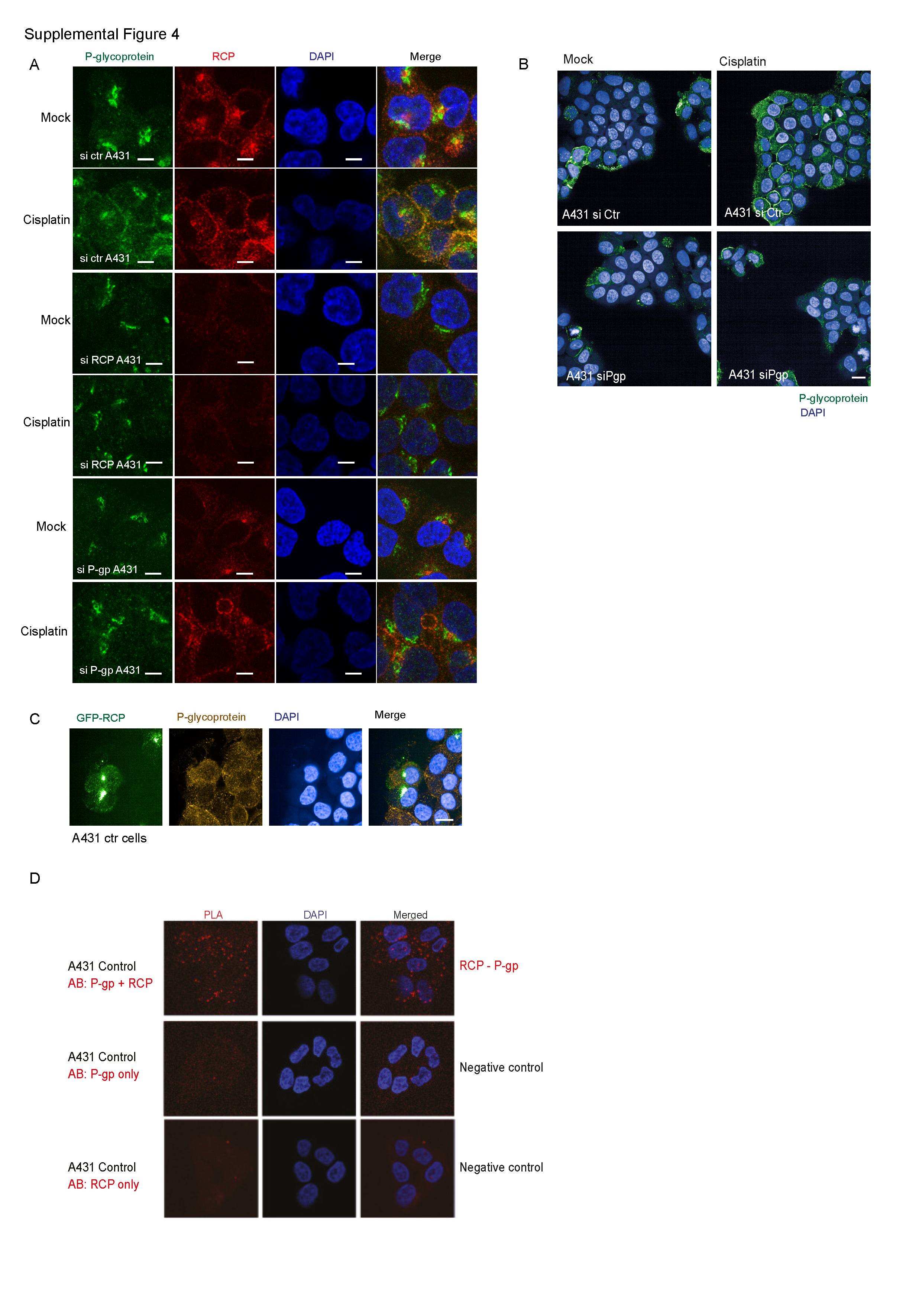

Supplement: Supplementary file 5 — Supplemental Figure 4. [file 41419_2021_3497_MOESM5_ESM.tif]

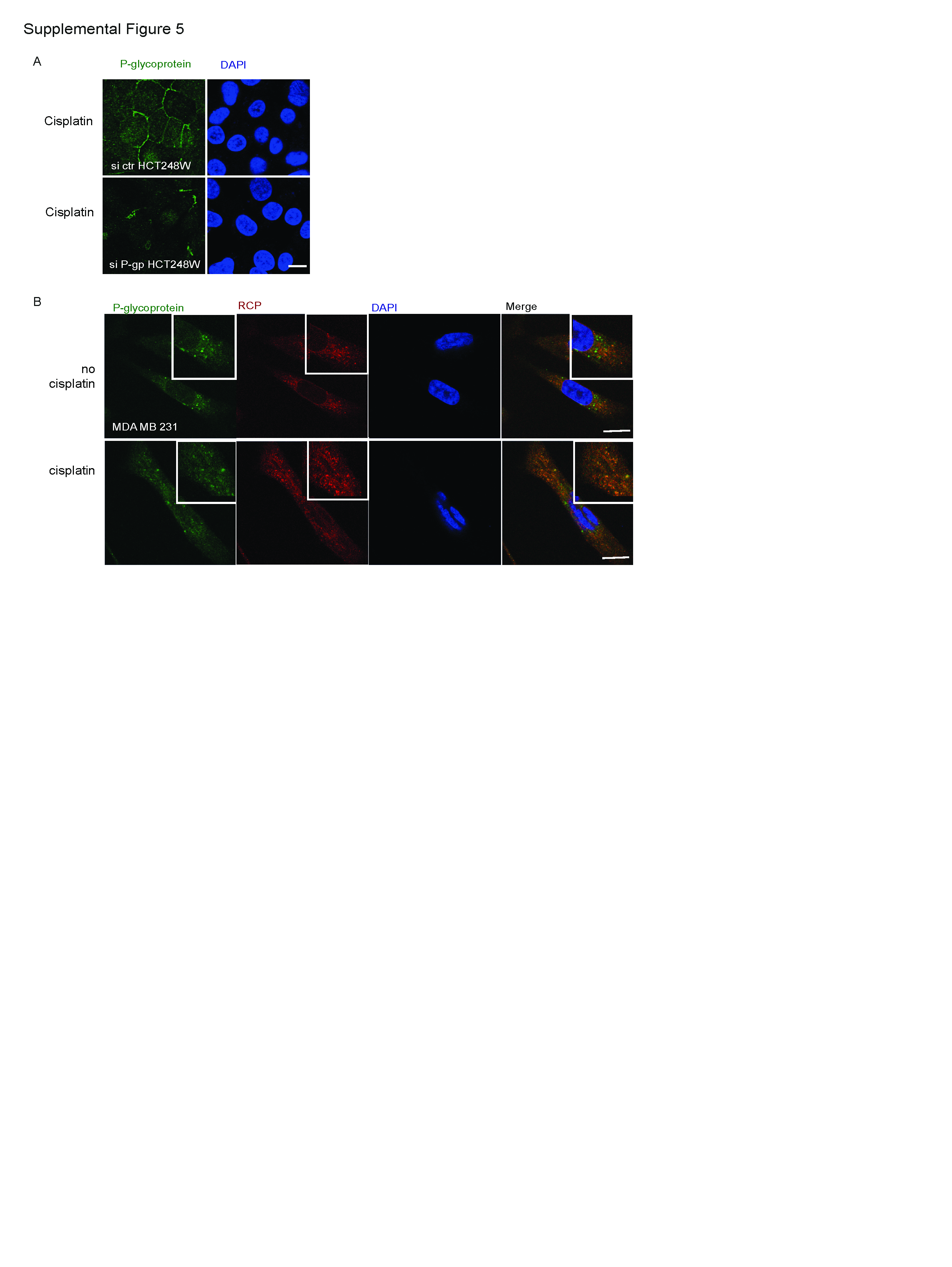

Supplement: Supplementary file 6 — Supplemental Figure 5. [file 41419_2021_3497_MOESM6_ESM.tif]

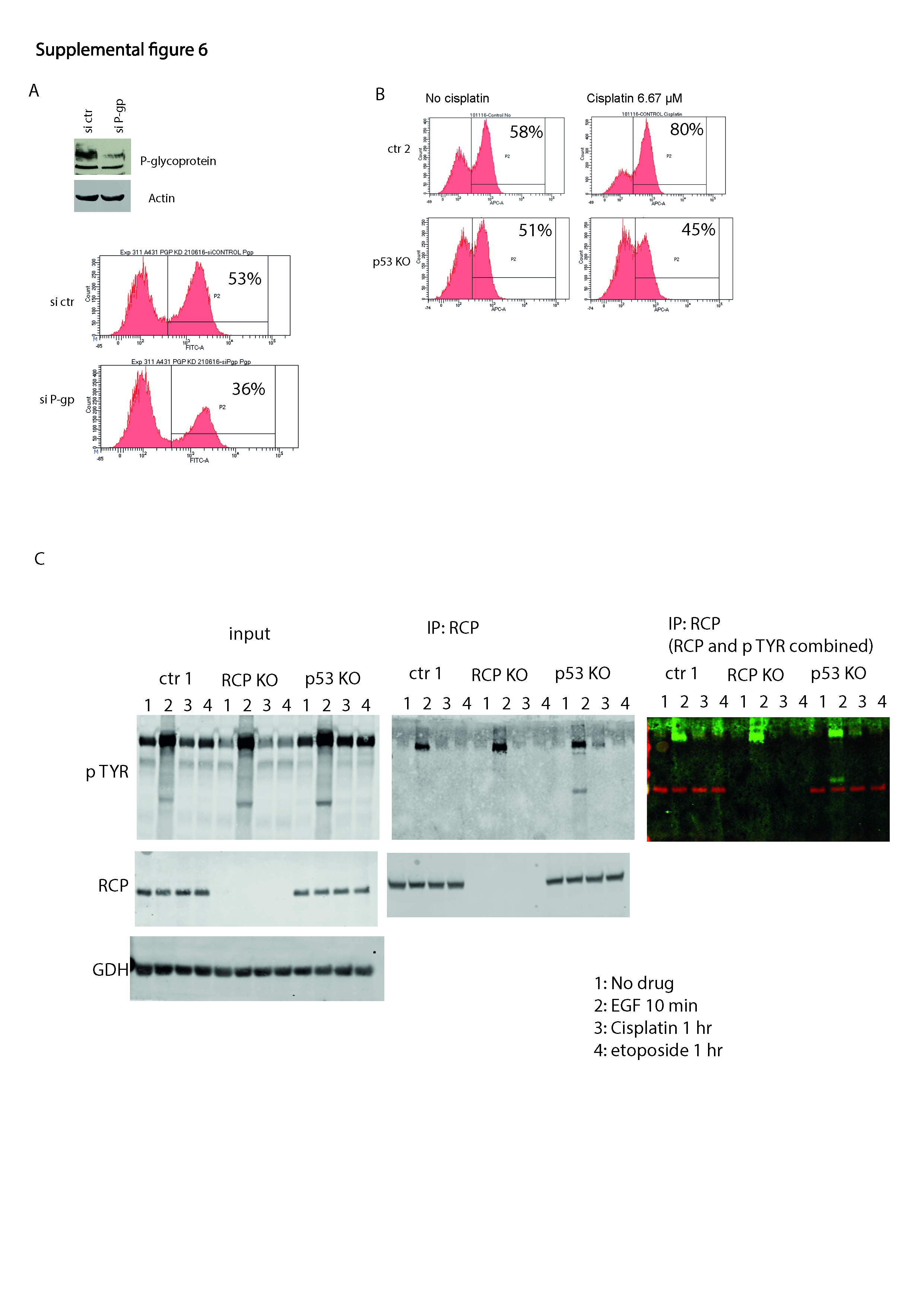

Supplement: Supplementary file 7 — Supplemental Figure 6. [file 41419_2021_3497_MOESM7_ESM.tif]
